# Diaphragm Dysfunction After Cardiac Surgery

**DOI:** 10.21470/1678-9741-2023-0239

**Published:** 2025-05-28

**Authors:** Tim Somers, Sandy Iskander, Ad F.T.M. Verhagen, Wilson W.L. Li

**Affiliations:** 1 Department of Cardiothoracic Surgery, Radboud University Medical Centre, Nijmegen, Netherlands

**Keywords:** Cardiac Surgery, Diaphragm Elevation, Phrenic Nerve Injury

## Abstract

**Introduction:**

Diaphragm elevation is commonly seen after cardiac surgery, mostly due to phrenic nerve injury. However, only historical data is available on the incidence of diaphragm elevation and its consequences during recovery.

**Objective:**

We aim to provide contemporary insights into the incidence of diaphragm dysfunction in patients undergoing cardiac surgery and its effect on postoperative outcomes.

**Methods:**

Records of all patients undergoing cardiac surgery through sternotomy between 2015 and 2016 at the Radboud University MedicalCentre were retrospectively reviewed. Diaphragm position and elevation were evaluated on available chest radiography. Right-sided diaphragm elevation was defined as the right diaphragm being > 3.0 cm above the left diaphragm; left-sided diaphragm elevation was defined as < 0.5 cm below or above the level of the right diaphragm.

**Results:**

A total of 1510 patients have undergone cardiac surgery through sternotomy during the study period, of which 1316 patients were included in the final analysis. Of these 1316 patients, 13% (n = 179) had pre-existing diaphragm elevation, 27% (n = 351) had a new diaphragm elevation postoperative-y, and 60% (n = 786) had no diaphragm elevation. No statistically significant differences were found between the groups in the occurrence of postoperative (pulmonary) complications or mortality. Of patients who developed new diaphragm elevation postoperatively, 65% recovered in the follow-up period.

**Conclusion:**

New postoperative diaphragm elevation occurs in 27% of patients undergoing cardiac surgery. However, new postoperative diaphragm elevation is not associated with a higher incidence of postoperative complications and spontaneous recovery is seen in most patients.

## INTRODUCTION

Diaphragm elevation caused by diaphragm dysfunction due to phrenic nerve injury is a well-recognized complication after cardiac surgery, with a reported incidence ranging from 1.2% to 60%^[1]^. Several technical risk factors associated with this phenomenon include internal mammary artery harvesting and cold injury of the phrenic nerve due to intrapericardial application of topical ice slush for myocardial protection^[1^, ^2^, ^3^, ^4^, ^5]^. Additionally, a higher incidence of diaphragm elevation is found in patients with chronic obstructive pulmonary disease (COPD) and/or diabetes mellitus^[6^, ^7]^. Diaphragm dysfunction can lead to adverse postoperative outcomes such as the need for prolonged mechanical ventilation^[8]^ atelectasis, and recurrent pneumonia^[9]^, as well as increased intensive care unit and hospital stay, morbidity, and mortality^[10]^.

However, reports on the incidence of diaphragm dysfunction and its consequences during recovery after cardiac surgery remain historical^[8]^. The aim of this study is to provide contemporary insights on the incidence of diaphragm dysfunction in patients undergoing cardiac surgery, its effect on postoperative outcomes, and the potential recovery of phrenic nerve injury during follow-up.

## METHODS

A retrospective cohort study was performed considering all patients who underwent cardiac surgery through sternotomy at the Radboud University Medical Centre (or Radboudumc) in Nijmegen, the Netherlands, between January 2015 and December 2016. Patients were excluded when death occurred during surgery, preoperative imaging was missing, postoperative imaging was missing, or pre and postoperative imaging could not be judged adequately (due to pleural effusion or atelectasis). This retrospective study was approved by the institutional review board (file number 2020-6728); no individual patient consent was required.

Data were obtained from digital patient charts and hospital registries and included detailed patient-, surgery-, and postoperative outcome-related information. The principal data used for the current analysis was based on the standardized Dutch National database of cardiac surgery (Begeleidingscommissie Hartinterventies Nederland [or BHN], a supervisory committee for heart interventions in the Netherlands) in which postoperative outcome parameters are prospectively being collected by the Department of Cardiothoracic Surgery.

In addition, diaphragm position and potential diaphragm elevation were evaluated on chest radiography (CR) at certain timepoints. Namely, the latest CR prior to surgery and the latest eligible CR prior to discharge but within a month after surgery. Right-sided diaphragm elevation was defined as the right diaphragm being > 3.0 cm above the left diaphragm^[11]^. Left-sided diaphragm elevation was defined as < 0.5 cm below or above the level of the right diaphragm. When available, follow-up imaging was evaluated as well to determine the occurrence of recovery in case of diaphragm elevation. In case of multiple follow-up images, the latest one was used for review. Possible follow-up outcomes were recovered elevation, persistent postoperative elevation, new elevation, or still no elevation present.

If no CR was available, other types of imaging were used when possible (*e. g,* computed tomography- or magnetic resonance imaging-scan). All CR were evaluated by the two main authors (SI, TS) independently. Disagreement was resolved by consensus, or after consultation with the final author (WWLL).

Patients were divided into three groups for statistical analyses: group A — pre-existing (hemi)diaphragm elevation, group B — new (hemi)diaphragm elevation, and group C — no (hemi) diaphragm elevation.

### Primary Endpoints

The primary endpoints were pulmonary complications (defined as pneumothorax with or without treatment, pleural effusion requiring drainage, pulmonary embolism, exacerbation of COPD, and/or special ventilatory requirements [ventilation in prone position]), in-hospital mortality, and recovery of new diaphragm elevation. Pulmonary complications combined with the need for reintubation formed our primary composite endpoint (composite 1).

### Secondary and Additional Endpoints

Secondary outcomes included the composite endpoint of pulmonary complications, reintubation, and in-hospital mortality (composite 2) and the composite endpoint of any form of complication (cardiac, pulmonary, renal, infectious, neurological complication, or reintubation) and/or in-hospital mortality after surgery (composite 3). Furthermore, when follow-up data was available, recovery of postoperative diaphragm elevations was evaluated.

### Statistical Analyses

All data was entered into an electronic database, Castor Electronic Data Capture (Castor EDC, Ciwit B.V., Amsterdam, the Netherlands), according to institutional regulations. Statistical analyses were performed using the software IBM SPSS Statistics for Windows, version 25.0 (Released 2017), Armonk, NY: IBM Corp. Continuous data are presented using means (± standard deviation). Categorical variables are presented with counts and percentages. Continuous data analysis was performed using the independent samples *t*-test, and categorical variables were compared using the chisquared test.

Multivariate logistic regression analysis was performed to determine whether change in diaphragm height difference is predictive of complications, in particular pulmonary complications. This change in diaphragm height was separated in groups of 2 cm, ranging from 0 to > 4.0 cm. Composite endpoints were formed to analyze the relationship between diaphragm elevation, respiratory complications, and mortality as previously defined. Multivariate logistic regression analysis was also performed to determine whether the presence of diaphragm elevation is predictive of complications. Interrater variability was compared using Pearson’s correlation coefficient. A P-value of < 0.05 was considered statistically significant.

## RESULTS

**Fig. 1:**
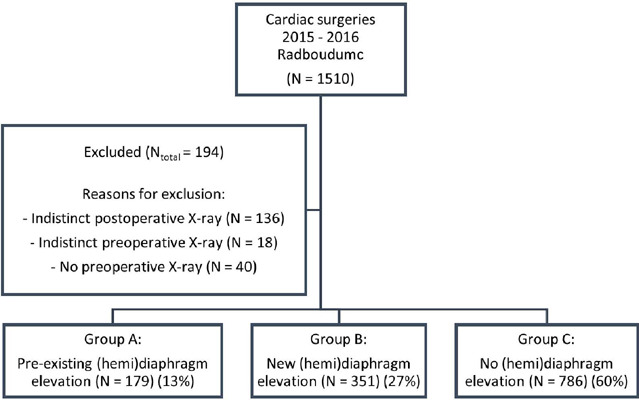
Research setting on diaphragm elevation after cardiac surgery. A flow diagram of the study depicting the number of excluded patients with their respected reasons and the three groups with pre-existent, new, and no (hemi) diaphragm elevation. Radboudumc=Radboud University Medical Centre.

A total of 1510 patients have undergone cardiac surgery through sternotomy during the study period (Figure 1). Of these, 12.8% (n = 194) were excluded, either due to missing preoperative imaging (n = 40), indistinct preoperative imaging (n = 18), or indistinct postoperative imaging (due to pleural effusion or atelectasis) (n = 136). This resulted in a total of 1316 patients included in the final analysis (Figure 1).

Of these 1316 patients, 13% (n = 179) had pre-existing diaphragm elevation (group A), 27% (n = 351) had a new diaphragm elevation postoperatively (group B), and 60% (n = 786) had no diaphragm elevation (group C). Of the patients with new postoperative diaphragm elevation, 64% (n = 223) had a left-sided elevation and 36% (n = 128) had right-sided elevation (*P* < 0.001). None of the patients had bilateral diaphragm elevation.

### Preoperative Demographic and Clinical Data

Baseline characteristics of patients for all three groups are presented in Table 1. For the total group, 74% were male, with a mean age of 65.87 ± 10.43 years. Patients in group A were significantly older than patients from groups B and C (A *vs.* B *P* = 0.008 and A *vs.* C *P* = 0.021). Additionally, patients from group A had significantly higher body mass index (BMI) when compared with those from group B (27.64 ± 3.94 *vs.* 26.83 ± 3.85, *P* = 0.023). Concerning preoperative comorbidities, more patients with diabetes were found in group C compared to group B (18% *vs.* 23%, *P* = 0.049). No other statistical differences were found in the baseline characteristics and clinical history.

**Table 1:** Patients’ characteristics.

	Total (N = 1316)	Group A (N = 179)	Group B (N = 351)	Group C (N = 786)	Overall P-value	P-value A *vs.* B	P-value A *vs.* C	P-value B *vs.* C
**Baseline characteristics**								
Age (years)	65.87 ± 10.43	67.73 ± 10.01	65.25 ± 10.21	65.73 ± 10.58	0.029	0.008	0.021	0.474
Male	976 (74%)	140 (78%)	265 (76%)	571 (73%)	0.247	0.487	0.111	0.308
Female	340 (26%)	39 (22%)	86 (25%)	215 (27%)				
BMI	27.05 ± 3.97	27.64 ± 3.94	26.83 ± 3.85	27.01 ± 4.02	0.076	0.023	0.055	0.495
**Preoperative conditions**								
Diabetes mellitus	282 (21%)	42 (24%)	62 (18%)	178 (23%)	0.130	0.125	0.814	0.049
Pulmonary disease	141 (11%)	15 (8%)	36 (10%)	90 (12%)	0.463	0.489	0.196	0.554
Previous cardiac surgery	77 (6%)	14 (8%)	14 (4%)	49 (6%)	0.159	0.092	0.438	0.098
Congenital heart disease	24 (2%)	3 (2%)	5 (1%)	16 (2%)	0.767	0.823	0.755	0.480
Radiotherapy	40 (3%)	4 (2%)	8 (2%)	28 (4%)	0.811	0.620	0.135	0.424
Infection	76 (6%)	13 (7%)	19 (5%)	44 (6%)	0.324	0.399	0.688	0.570
Immunological disease	36 (3%)	4 (2%)	11 (3%)	21 (3%)	0.167	0.556	0.594	0.947
**Type of previous cardiac surgery**					0.990	1.000	0.912	0.908
Valve surgery	19 (1%)	3 (2%)	4 (1%)	12 (2%)				
CABG/OPCAB	15 (1%)	3 (2%)	3 (1%)	9 (1%)				
Valve surgery + CABG	4 (0.3%)	0 (0%)	0 (0%)	4 (0.5%)				
Aortic surgery	20 (2%)	4 (2%)	4 (1%)	12 (2%)				
Various	20 (2%)	4 (2%)	4 (1%)	12 (2%)				

Baseline characteristics of all study patients and separated for the three groups with pre-existing (hemi)diaphragm elevation (group A), new (hemi)diaphragm elevation (group B), and no (hemi)diaphragm elevation (group C). Results are depicted as mean ± standard deviation or as absolute numbers with percentages. *P*-values < 0.05 are deemed statistically significant

BMI=body mass index; CABG=coronary artery bypass grafting; OPCAB=off-pump coronary artery bypass grafting

### Type of Surgery

Regarding the effect of the type of cardiac surgery on the incidence of new diaphragm elevation postoperatively (Table 2), we found that patients were most often affected after aortic surgery (37%), however this difference was not statistically significant. No statistically significant differences were observed between the other different types of surgery, nor between cases using left internal mammary artery (LIMA) and/or right internal mammary artery (RIMA) in the coronary artery bypass grafting (CABG) or off-pump coronary artery bypass grafting.

**Table 2:** The incidence of diaphragm elevation within each type of surgery.

	Type of surgery							
(Hemi)diaphragm elevation	Total (N = 1316)	Valve surgery (N = 313)	CABG/OPCAB (N = 694)	Valve surgery + CABG (N = 132)	Aortic surgery (N = 128)	Various (N = 49)	LIMA (N = 765)	RIMA (N = 94)
A – Pre-existing	179 (14%)	38 (12%)	84 (12%)	32 (24%)	18 (14%)	7 (14%)	106 (14%)	13 (14%)
B – New	351 (27%)	88 (28%)	177 (26%)	28 (21%)	47 (37%)	11 (22%)	190 (25%)	18 (19%)
C – No	786 (60%)	187 (60%)	433 (62%)	72 (55%)	63 (49%)	31 (63%)	469 (61%)	63 (67%)

Absolute number of pre-existing, new, or no (hemi)diaphragm elevation with percentages separated for each type of surgery performed. In case of CABG, the use of LIMA or RIMA is shown for all three groups

CABG=coronary artery bypass grafting; LIMA=left internal mammary artery; OPCAB=off-pump coronary artery bypass grafting; RIMA=right internal mammary artery

### Postoperative Outcomes and Follow-up

As seen in Table 3, infectious and cardiac complications occurred most frequently (7-8%), whereas renal complications and hospital mortality occurred the least (1-2%). No statistically significant differences were found between the groups. Analysis on hospital mortality found no relationship between the groups and outcome. There were no statistically significant differences regarding the composite endpoints between the three groups (Table 3).

**Table 3:** Postoperative outcomes.

	Total (N = 1316)	Group A (N = 179)	Group B (N =351)	Group C (N = 786)	Overall *P*-value	P-value A *vs.* B	P-value A *vs.* C	P-value B *vs.* C
Postoperative complications								
Cardiac complications	105 (8%)	14 (8%)	25 (7%)	65 (8%)	0.762	0.771	0.801	0.465
Heart rhythm problems other than atrial fibrillation requiring pacemaker		4 (4%)	5 (5%)	14 (13%)				
Infarction		1 (1%)	1 (1%)	6 (6%)				
Resuscitation (due to conduction disorder *vs.* other)		0 *vs.* 1 (1%)	2 *vs.* 1 (3%)	2 *vs.* 2 (4%)				
Pericardiocentesis		2 (2%)	1 (1%)	9 (9%)				
Subxiphoid drainage		1 (1%)	3 (3%)	5 (5%)				
Re-sternotomy		5 (5%)	12 (11%)	26 (25%)				
Reoperation		0	0	1 (1%)				
ECMO in right ventricular failure		0	0	0				
Pulmonary complications	53 (4%)	6 (4%)	13 (4%)	33 (4%)	0.923	0.906	0.862	0.696
Pneumothorax, no treatment required		0	3 (6%)	1 (2%)				
Pneumothorax requiring drainage		0	6 (11%)	14 (26%)				
Pleural effusion requiring drainage		2 (4%)	3 (6%)	10 (19%)				
Pulmonary embolism		0	0	1 (2%)				
Exacerbation of COPD		2 (4%)	0	4* (8%)				
Special ventilatory requirements (abdominal breathing support)		2 (4%)	1 (2%)	5^#^ (9%)				
Renal complications	23 (2%)	4 (2%)	7 (2%)	12 (2%)	0.743	0.855	0.504	0.570
AKI		2 (9%)	5 (22%)	10 (43%)				
Requiring CVVH		2 (9%)	2 (9%)	2 (9%)				
Infectious complications	87 (7%)	13 (7%)	24 (7%)	51 (7%)	0.975	0.954	0.916	0.827
HAP		9 (10%)	15^!^ (16%)	30 (34%)				
UTI		3 (3%)	3 (3%)	13 (15%)				
Superficial wound infection		0	0	2 (2%)				
Mediastinitis		0	2 (2%)	2^$^ (1%)				
Other (*e. g,* bacteremia, leg wound infection)		2 (2%)	5 (6%)	5^$^ (5%)				
Neurological complications	35 (3%)	7 (4%)	8 (2%)	20 (3%)	0.614	0.326	0.533	0.572
CVA		6 (18%)	3 (9%)	12 (34%)				
Bleeding		0	1 (3%)	1 (3%)				
TIA		0	0	4 (11%)				
Spinal cord ischemia		0	1 (3%)	0				
Other (*e. g.,* recurrent lesion, epilepsy, or ICU acquired weakness)		1 (3%)	3 (9%)	3 (9%)				
Reintubation	30 (2%)	7 (4%)	9 (3%)	14 (2%)	0.208	0.393	0.165	0.387
Mechanical ventilation duration, days)	0.78 ± 2.73	0.63 ± 1.07	0.91 ± 3.83	0.76 ± 2.38	0.486	0.327	0.471	0.407
ICU stay, days	2.04 ± 5.27	1.91 ± 3.00	1.90 ± 4.08	2.13 ± 6.09	0.752	0.976	0.643	0.524
Hospital stay, days	6.98 ± 8.09	6.82 ± 5.025	6.66 ± 6.82	7.16 ± 9.12	0.611	0.788	0.630	0.364
**Mortality**								
Hospital mortality	11 (1%)	3 (2%)	1 (0.3%)	7 (1%)	0.242	0.167	0.350	0.169
**Composite endpoint 1**	71	9	21	41	0.160	0.232	0.382	0.533
**Composite endpoint 2**	78	11	22	45	0.403	0.340	0.368	0.731
**Composite endpoint 3**	242	34	60	148	0.414	0.492	0.943	0.295

The postoperative outcomes and complications for all study patients and separated for the three groups with pre-existing (hemi) diaphragm elevation (group A), new (hemi)diaphragm elevation (group B), and no (hemi)diaphragm elevation (group C). Results are depicted as mean ± standard deviation or as absolute numbers with percentages. Composite endpoint 1 = pulmonary complication + reintubation; composite endpoint 2 = pulmonary complication + reintubation + hospital mortality; composite endpoint 3 = all complications together. *P*-values < 0.05 are deemed statistically significant

*one patient who also had a pneumothorax requiring drain; ^#^one patient who also had pleural drainage; ^!^one patient who also had urinary tract infection; ^$^one patient who also had pneumonia

AKI=acute kidney injury; COPD=chronic obstructive pulmonary disease; CVA=cerebrovascular accident; CVVH=continuous veno-venous hemofiltration; ECMO=extracorporeal life support; HAP=hospital-acquired pneumonia; ICU=intensive care unit; TIA=transient ischemic attack; UTI=urinary tract infection

In multivariate analysis (Table 4), neither newly developed diaphragm elevation (odds ratio [OR] 1.087, 95% confidence interval [CI] 0.622-1.901, *P* = 0.769 and OR 1.046, 95% CI 0.607-1.803, *P* = 0.871, for composites 1 and 2, respectively) nor postoperative diaphragm elevation in centimeters (OR 1.539, 95% CI 0.841-2.817, *P* = 0.162 and OR 1.062, 95% CI 0.356-3.169, *P* = 0.914; OR 1.344, 95% CI 0.739-2.445, *P* = 0.332 and 0.854 95% CI 0.287-2.534, *P* = 0.775, respectively 2-4 cm and > 4 cm for composites 1 and 2) were significant predictors for composite endpoint 1, or for composite endpoint 2.

**Table 4:** Logistic regression analyses.

Variables	Odds ratio	Standard error	*P*-value	95% CI
Composite 1 *vs.* difference in height in groups of 2 cm				
Sex	0.812	0.286	0.468	0.464 – 1.423
Age (years)	1.014	0.012	0.250	0.990 – 1.039
BMI	0.974	0.032	0.408	0.915 – 1.037
Pulmonary disease	2.553	0.311	0.003	1.388 – 4.694
Diabetes mellitus	1.408	0.321	0.287	0.750 – 2.642
Previous thoracic/cardiac surgery	0.946	0.444	0.900	0.396 – 2.259
Type of surgery				
CABG	1	1	< 0.001	1
OPCAB	1.447	1.073	0.730	0.177 – 11.850
Valve surgery	2.181	0.345	0.024	1.109 – 4.290
Valve + CABG	1.714	0.461	0.243	0.694 – 4.233
Aorta	6.387	0.384	< 0.001	3.010 – 13.551
0-2 cm height difference	1	1	0.375	1
2-4 cm height difference	1.539	0.308	0.162	0.841 – 2.817
> 4 cm height difference	1.062	0.558	0.914	0.356 – 3.169
Composite 1 *vs.* presence of diaphragm elevation				
Sex	0.795	0.286	0.422	0.454 – 1.393
Age (years)	1.014	0.012	0.257	0.990 – 1.038
BMI	0.976	0.032	0.441	0.916 – 1.039
Pulmonary disease	2.552	0.310	0.003	1.389 – 4.688
Diabetes mellitus	1.407	0.321	0.288	0.750 – 2.640
Previous thoracic/cardiac surgery	0.958	0.445	0.923	0.400 – 2.292
Type of surgery				
CABG	1	1	< 0.001	1
OPCAB	1.574	1.067	0.671	0.195 – 12.732
Valve surgery	2.213	0.344	0.021	1.127 – 4.348
Valve + CABG	1.783	0.460	0.209	0.723 – 4.397
Aorta	6.734	0.380	< 0.001	3.198 – 14.180
No elevation	1	1	0.865	1
New elevation	1.087	0.285	0.769	0.622 – 1.901
Pre-existing elevation	0.867	0.391	0.714	0.403 – 1.864
Composite 2 *vs.* difference in height in groups of 2 cm				
Sex	0.879	0.271	0.634	0.517 – 1.494
Age (years)	1.023	0.012	0.060	0.999 – 1.048
BMI	0.979	0.031	0.479	0.922 – 1.039
Pulmonary disease	2.432	0.302	0.003	1.345 – 4.397
Diabetes mellitus	1.588	0.301	0.124	0.881 – 2.862
Previous thoracic/cardiac surgery	1.026	0.421	0.951	0.449 – 2.345
Type of surgery				
CABG	1	1	< 0.001	1
OPCAB	1.348	1.073	0.781	0.165 – 11.035
Valve surgery	2.323	0.333	0.011	1.210 – 4.462
Valve + CABG	2.100	0.421	0.078	0.920 – 4.793
Aorta	7.498	0.372	< 0.001	3.619 – 15.536
0-2 cm height difference	1	1	0.575	1
2-4 cm height difference	1.344	0.305	0.332	0.739 – 2.445
> 4 cm height difference	0.854	0.555	0.775	0.287 – 2.534
Composite 2 *vs.* presence of diaphragm elevation				
Sex	0.864	0.271	0.590	0.508 – 1.470
Age (years)	1.023	0.012	0.067	0.998 – 1.047
BMI	0.980	0.031	0.501	0.922 – 1.040
Pulmonary disease	2.437	0.302	0.003	1.349 – 4.403
Diabetes mellitus	1.579	0.300	0.128	0.876 – 2.845
Previous thoracic/cardiac surgery	1.045	0.422	0.916	0.457 – 2.392
Type of surgery				
CABG	1	1	< 0.001	1
OPCAB	1.427	1.069	0.740	0.176 – 11.588
Valve surgery	2.324	0.333	0.011	1.211 – 4.459
Valve + CABG	2.114	0.421	0.076	0.926 – 4.825
Aorta	7.599	0.368	< 0.001	3.695 – 15.630
No elevation	1	1	0.966	1
New elevation	1.046	0.278	0.871	0.607 – 1.803
Pre-existing elevation	0.944	0.361	0.873	0.466 – 1.914

Logistic regression analyses to determine the association between change in diaphragm height (top panel) and the presence of diaphragm elevation (bottom panel) for pulmonary complication and reintubation (composite 1) and pulmonary complication with reintubation and mortality (composite 2). *P*-values < 0.05 are deemed statistically significant BMI=body mass index; CABG=coronary artery bypass grafting; CI=confidence interval; OPCAB=off-pump coronary artery bypass grafting

Almost a third of all patients had follow-up imaging available (n = 404), of which the results are shown in Table 5. The median follow-up time is 17.5 months (range 0 - 58 months). Interestingly, as seen in Table 6, 65% of patients who developed new diaphragm elevation postoperatively recovered in the follow-up period. Of all surgical interventions, patients who underwent aortic surgery most often had imaging available in the follow-up period (93% *vs.* < 50% for all other interventions).

**Table 5:** Follow-up (patient) characteristics.

Variables	With follow-up data	Without follow-up data	*P*-value
Total number of patients	404	912	
% Female	32.7% (n = 132)	22.8% (n = 208)	< 0.001
Age (years)	62.6 ± 11.8	67.3 ± 9.4	< 0.001
BMI	26.9 ± 4.3	27.1 ± 3.8	0.458
Pulmonary disease	57 (14.1%)	84 (9.2%)	0.008
Diabetes mellitus	68 (16.8%)	214 (23.5%)	0.007
Previous cardiac surgery	47 (11.6%)	30 (3.3%)	<0.001
CABG	7 (1.7%)		
Valve	15 (3.7%)		
Aortic	14 (3.5%)		
Congenital heart disease	19 (4.7%)	5 (0.5%)	< 0.001
Previous radiotherapy	18 (4.5%)	22 (2.4%)	0.072
Current type of surgery			
CABG/OPCAB	137 (33.9%)	557 (61.1%)	
Valve	92 (22.8%)	221 (24.2%)	
Valve + CABG	33 (8.2%)	99 (10.9%)	
Aorta	119 (29.5%)	9 (1.0%)	
Other	23 (5.7%)	26 (2.9%)	
Days at ICU	2.9 ± 7.0	1.6 ± 4.3	0.001
Days of intubation	1.2 ± 4.4	0.6 ± 1.4	0.004
Reintubation	16 (4.0%)	14 (1.5%)	0.007
Days in hospital	10.1 ± 11.7	5.6 ± 5.2	0.000
Mortality	1 (0.2%)	10 (1.1%)	0.119
Cardiac complications	54 (13.4%)	50 (5.5%)	< 0.001
Pulmonary complications	26 (6.4%)	26 (2.9%)	0.002
Renal complications	12 (3.0%)	11 (1.2%)	0.024
Postoperative infection	54 (13.4%)	34 (3.7%)	< 0.001
Neurological complications	23 (5.7%)	12 (1.3%)	< 0.001
Postoperative change in diaphragm height			0.013
< 2 cm	312 (77.2%)	753 (82.6%)	
2 - 4 cm	67 (16.6%)	131 (14.4%)	
> 4 cm	25 (6.2%)	28 (3.1%)	
Elevation in follow-up			
No elevation (total)	281 (69.6%)	0	-
Left	81 (20.0%)		
Right	42 (10.4%)		
Recovery during follow-up			
No elevation	189 (46.8%)		-
Newly developed elevation	48 (11.9%)	0	
Persistent elevation	75 (18.6%)		
Elevation recovered	92 (22.8%)		
Months of follow-up	17.5 (range 0 - 58)	0	-

Characteristics of all study patients separated for presence or absence of follow-up data. Results are depicted as mean ± standard deviation, as absolute numbers with percentages or as median with range. *P*-values < 0.05 are deemed statistically significant

BMI=body mass index; CABG=coronary artery bypass grafting; ICU=intensive care unit; OPCAB=off-pump coronary artery bypass grafting

**Table 6:** Follow-up for group B (new elevation).

	Group B (N = 351)
**Follow-up**	
Persistent elevation	41 (35%)
New elevation present	0
Still no elevation	0
Elevation recovered	76 (65%)
**Total**	117 (33%)
Months of follow-up	16.0 (0 - 55)

Characteristics of patients that were assigned to the group with new postoperative (hemi)diaphragm elevation (group B) of whom follow-up data was present. Results are depicted as absolute numbers with percentages or a median with a range

### Interrater Reliability

For 240 randomly chosen patients, pre, postoperative, and, when available, follow-up diaphragm distances have been measured by two observers. This has resulted in 542 pairs of data. The mean difference between these measurements was 0.0304 cm (limits of agreement = 0.030 +/- 1.96 × 0.123). Using Pearson’s *r* test, a correlation coefficient of 0.939 (*P* < 0.001) was found. This indicates a very strong level of agreement between both observers.

## DISCUSSION

Elevation of the diaphragm is a known complication after cardiothoracic surgery. This study presents that around a quarter of all patients (27%) suffers from new postoperative diaphragm elevation, regardless the type of surgery, although the incidence is seen after aortic surgery. No significant differences were found in postoperative outcomes. Neither was there a positive correlation between the level of diaphragm shift relative to each other and any of the composite endpoints. Strikingly, almost three quarters of all patients who develop diaphragm elevation post cardiac surgery recovered from this elevation in the months after discharge. However, when comparing both the group with follow-up data and the group without it, it is found that the difference in demographic data is statistically significant, meaning that the group with follow-up data might not be representative for the entire population. This could be clarified by the fact that patients undergoing aortic surgery have a much more stringent follow-up protocol including imaging compared to the patients undergoing standard CABG or valve surgery. The aortic surgery patients were also most affected, although not significantly, by new diaphragm elevation.

As mentioned before, the reported incidence of diaphragm elevation ranges between 2% and 60%^[2^, ^10^, ^12^, ^13^, ^14]^. Most of these studies present historical data, with the most recent articles on diaphragmatic dysfunction after cardiac surgery with significant numbers published between 1994 and 2001^[8^, ^15^, ^16]^. Our study, using contemporary data, reports an incidence of new postoperative diaphragm elevation of 27%, meaning that one in four patients undergoing cardiac surgery will suffer from (often temporary) diaphragm elevation. This high number is a consequence of the way diaphragm dysfunction was evaluated. As stated by Chetta et al.^[17]^, CR is not suitable for predicting diaphragm function, but it is a great tool for determining the height of the diaphragm and the corresponding diaphragmatic height index^[18]^. As such, it is still the most commonly used first step in diagnosing possible diaphragm dysfunction^[19]^.

The number of patients with (new) diaphragm elevation is of course dependent on the cutoff value used to define diaphragm elevation. Many agree upon the left diaphragm elevation if it is at the level of the right or above, however for the right hemidiaphragm this is less distinct. This study used 3 cm as cutoff point, as this is commonly described in the literature^[11^, ^20^, ^21]^. However, other studies suggest lower values, which would further increase the incidence numbers of diaphragm elevation and could be an explanation for the wide range in the previously described incidence numbers^[19]^. Previous studies mainly focus on the presence of diaphragm elevation using different definitions^[1^, ^2^, ^17^, ^18^, ^19]^. However, most of the new diaphragm elevations were minor changes (< 2 cm), which could explain why no significant difference was seen between the pre-existent and no elevation group.

Most new diaphragm elevations were seen in patients undergoing aortic surgery. This could be related to the use of topical ice slush in our centre. Multiple studies have already shown that the use of ice slush for topical hypothermia in cardiac surgery is associated with diaphragm paralysis^[5^, ^22]^. Since it was shown to have no additional benefit, topical ice should be discouraged^[23]^. In most cases, this is a transient paresis^[24]^, as described in our study with a 65% recovery rate of patients with new postoperative diaphragm elevation. Therefore, in case of new diaphragm dysfunction after cardiac surgery, either “wait and see” or intensive physiotherapy can be initiated in the early postoperative phase. More definite injury to the phrenic nerve is seen in CABG when using the cautery in close proximity to the nerve during harvesting of LIMA and/or RIMA^[2^, ^25]^. Diaphragm plication is the proposed therapy in this setting of persistent diaphragm elevation combined with significant complaints of pulmonary deterioration^[19]^. Although early spontaneous recovery is rare, previous studies confirm spontaneous recovery in over half of patients on the long term^[2^, ^26^, ^27]^.

### Limitations

A limitation of this study was the use of radiographic technique. Atelectasis or pleural effusion complicated the analysis of exact diaphragm heights resulting in certain patients being excluded, even though a suitable number of patients remained to be included for analyses. Also, in case of diaphragm elevation, this was not confirmed by functional imaging (ultrasound or fluoroscopy). However, the retrospective character of this study and the limited availability of functional imaging hampered more detailed evaluation. Another issue is the loss to follow-up. As this study was performed in a tertiary referral centre, many patients had follow-up in another hospital.

## CONCLUSION

Diaphragm elevation is a complication that occurs frequently after cardiac surgery. However no significant correlation was found between diaphragm elevation, the distance in diaphragm height, and the outcomes after surgery. In most cases, the elevation recovers spontaneously. Future directions should focus on a larger number of patients with longer follow-up and functional testing as well as the consideration of slushed ice and use of cautery in CABG as a risk factor to explore amendments required for clinical practice.

## Abbreviations, Acronyms & Symbols

AKI: Acute kidney injury
BMI: Body mass index
CABG: Coronary artery bypass grafting
CI: Confidence interval
COPD: Chronic obstructive pulmonary disease
CR: Chest radiography
CVA: Cerebrovascular accident
CVVH: Continuous veno-venous hemofiltration
ECMO: Extracorporeal life support
HAP: Hospital-acquired pneumonia
ICU: Intensive care unit
LIMA: Left internal mammary artery
OPCAB: Off-pump coronary artery bypass grafting
OR: Odds ratio
RIMA: Right internal mammary artery
TIA: Transient ischemic attack
UTI: Urinary tract infection
